# Next-Generation Energy Storage Materials Explored by Advanced Scanning Techniques

**DOI:** 10.1155/2018/3280283

**Published:** 2018-12-03

**Authors:** Huaiyu Shao, Hai-Wen Li, Ya-Jun Cheng, Huaijun Lin, Liqing He

**Affiliations:** ^1^Institute of Applied Physics and Materials Engineering (IAPME), University of Macau, Macau; ^2^Kyushu University, Fukuoka, Japan; ^3^Ningbo Institute of Materials Technology and Engineering, Chinese Academy of Sciences, Ningbo, China; ^4^University of Oxford, Oxford, UK; ^5^Jinan University, Guangzhou, China; ^6^Southern University of Science and Technology, Shenzhen, China

Energy storage, which is capturing and storing energy produced at one time and/or at a certain place for use at a later time and/or other locations, is one of the most critical issues for human society to realize sustainability. Development of next-generation energy storage materials is one of the hottest research topics in the materials science field. In recent decades, advanced scanning techniques including SEM, TEM, AFM, STMs, and Raman spectroscopy have been abundantly employed in observing morphologies, characterizing microstructures, and identifying specific physical and chemical properties in order to design innovative materials with controllable structures, understand the formation mechanisms, clarify the catalytic mechanisms, and elucidate the effect of designed parameters on energy storage properties [1]. The aim of this special issue is to publish high-quality research papers as well as provide a comprehensive review addressing the latest and state-of-the-art topics from active researchers in the field of energy storage materials. This special issue contains 10 research papers and 1 review paper representing some of the latest research in energy storage materials explored by advanced scanning techniques.

Hydrogen storage materials are one of the main research topics in this special issue. Mg-based materials have attracted great attention because of the interest in high-capacity hydrogen storage applications in the past decades [2, 3]. J. Li et al. reviewed the advanced SEM and TEM techniques applied in Mg-based hydrogen storage research. The reviewed literature implies that the applications of advanced SEM and TEM play significantly important roles in the research and development of next-generation hydrogen storage materials. H. He et al. reported the structural and hydrogenation kinetic properties of a Zr_0.8_Ti_0.2_Co alloy prepared by ball milling. High-resolution- (HR-) TEM studies revealed that a large number of disordered microstructures including amorphous regions and defects existed after ball milling, and these played an important role in improving the hydrogenation performances. B. Li et al. synthesized FCC-structure TiVMn-based and TiCrMn-based nanoalloys with a mean particle size of around a few to tens of *μ*m and with an average crystallite size of just 10 to 13 nm. The microstructures of the alloys were carefully studied by SEM and XRD, while hydrogen storage properties were studied by high-pressure DSC under a H_2_ atmosphere. It showed that the absorption reaction was much stronger, and it started at a much lower temperature (210°C) in the TiVMn nanoalloy than that in the TiCrMn one.

Studies on rechargeable lithium-ion batteries (LIBs) [4] and sodium-ion batteries (SIBs) [5] have become one of the most widely investigated research directions all over the world, and they are also the main topics included in this special issue. LIBs currently have governed the worldwide rechargeable battery markets due to their outstanding energy and power capability. In recent days, research interest in SIBs has been resurrected, driven by new applications with requirements different from those in portable electronics and the need to address the concern of low Li abundance in the Earth's crust. SnO_2_ has been considered as an outstanding alternative to graphite as an anode for LIBs [6]. P. Yu et al. synthesized SnO_2_ nanoparticles by a novel route of the sol-gel method assisted with biomimetic assembly using L-leucine as a biotemplate. The results demonstrated that the growth of SnO_2_ nanoparticles could be regulated by L-leucine at a high calcination temperature. D. Cui et al. reported that the LiNi_0.8_Co_0.15_Al_0.05_O_2_/graphite LIBs showed better cycle lives at a 2.0 C discharge rate than that at a 1.5 C discharge rate, which was due to the reason that the negative electrodes contributed more than the positive electrodes. Q. Sun et al. reported a novel open-framework Cu-Ge-based chalcogenide, [Cu_8_Ge_6_Se_19_](C_5_H_12_N)_6_ (CGSe), as an anode material for SIBs. As a result, the CGSe anode exhibited good electrochemical performances such as high reversible capacity (463.3 mAh g^−1^), excellent rate performance, and considerable cycling stability. X. Zhang et al. synthesized flexible freestanding carbon nanofiber-embedded TiO_2_ nanoparticles (CNF-TiO_2_) and then applied it directly as the anode in SIBs without a binder or current collector. The anode exhibited a high reversible capacity of 614 mAh g^−1^ (0.27 mAh cm^−2^) after almost 400 cycles and an excellent capacity retention ability of ~100%.

Supercapacitors have drawn great attention due to their high power, energy density and long lifecycle [7]. J. Cui et al. designed and fabricated Ag-ion-modified titanium nanotube (Ag/TiO_2_-NT) arrays as the electrode material of supercapacitors for electrochemical energy storage. The modified electrode showed a high capacitance of 9324.6 mF·cm^−3^ (86.9 mF·g, 1.2 mF·cm^−2^), energy density of 82.8 *μ*Wh·cm^−3^ (0.8 *μ*Wh·g, 0.0103 *μ*Wh·cm^−2^), and power density of 161.0 mW·cm^−3^ (150.4 *μ*W·g, 2.00 *μ*W·cm^−2^) at the current density of 0.05 mA.

Exploring earth-abundant and cost-effective catalysts with high activity and stability for hydrogen evolution reaction (HER) is of great importance to practical applications of alkaline water electrolysis [8]. X. Li et al. reported on A-site Ba^2+^-deficiency doping as an effective strategy to enhance the electrochemical activity of BaCo_0.4_Fe_0.4_Zr_0.1_Y_0.1_O_3−*δ*_ for HER, which was related to the formation of oxygen vacancies around active Co/Fe ions.

The heterojunction system has been proven to be one of the best architectures for photocatalysts [9]. L. Han et al. reported the facile synthesis of indium sulfide/flexible electrospun carbon nanofiber (In_2_S_3_/CNF) for enhanced photocatalytic efficiency. The prepared In_2_S_3_/CNF photocatalysts exhibited greatly enhanced photocatalytic activity compared to pure In_2_S_3_. In addition, the formation mechanism of the one-dimensional heterojunction In_2_S_3_/CNF photocatalyst was discussed.

Congo red 1-naphthalenesulfonic acid is the critical source of contamination of wastewater [10]. P. Yu et al. reported a composite of pyrolytic Triarrhena biochar loading with TiO_2_ nanoparticles synthesized by a sol-gel method. When used as an absorbent to remove Congo red from an aqueous solution, it was found that the as-prepared composite performed better absorption capacity than a single biochar or TiO_2_.

In summary, the contributed papers in this special issue cover several general aspects of energy storage materials, which are explored by scanning techniques, including SEM, TEM, AFM, STMs, and Raman spectroscopy. The advancement of scanning technologies requires deeply understanding the microstructures of energy materials and elucidating the mechanisms of material properties with various structures. It may then bring possible breakthroughs in the development of next-generation energy storage materials. We tried our best to present the latest cutting-edge applications of scanning techniques in this field and hope that our endeavor may shed light on future research on energy storage technologies.
